# Supplementary material for: Analyzing Citramalic Acid Enantiomers in Apples and Commercial Fruit Juice by Liquid Chromatography–Tandem Mass Spectrometry with Pre-Column Derivatization
Source: Molecules. 2023 Feb 6;28(4):1556. doi: 10.3390/molecules28041556 (PMC9959191; doi:10.3390/molecules28041556)
Supplement: Supplementary file 1 [file molecules-28-01556-s001.zip › molecules-2173674-supplementary.pdf]

Supporting information

Analyzing citramalic acid enantiomers in apple and commercial fruit juice by liquid chromatography–tandem mass spectrometry with pre-column derivatization

Maho Umino, Mayu Onozato, Tatsuya Sakamoto, Mikoto Koishi, Takeshi Fukushima*

Department of Analytical Chemistry, Faculty of Pharmaceutical Sciences, Toho University

2-2-1 Miyama, Funabashi-shi, Chiba 274-8510, Japan

*Takeshi Fukushima (Corresponding author): t-fukushima@phar.toho-u.ac.jp

Faculty of Pharmaceutical Sciences, Toho University,

2-2-1 Miyama, Funabashi-shi, Chiba 274-8510, Japan

Phone number/Fax: 81-47-472-1504

Contents

Scheme S1. Biosynthetic pathways of (a) *R*- and (b) *S*-CMA. ・・・・・ Page 3

Figure S1. MS/MS spectra of *R*-CMA detected in samples prepared from apple peel, fruit, and juice. ・・・・・ Page 4

Figure S2. MRM chromatogram of the internal standard used in the present study. ・・・・・ Page 5

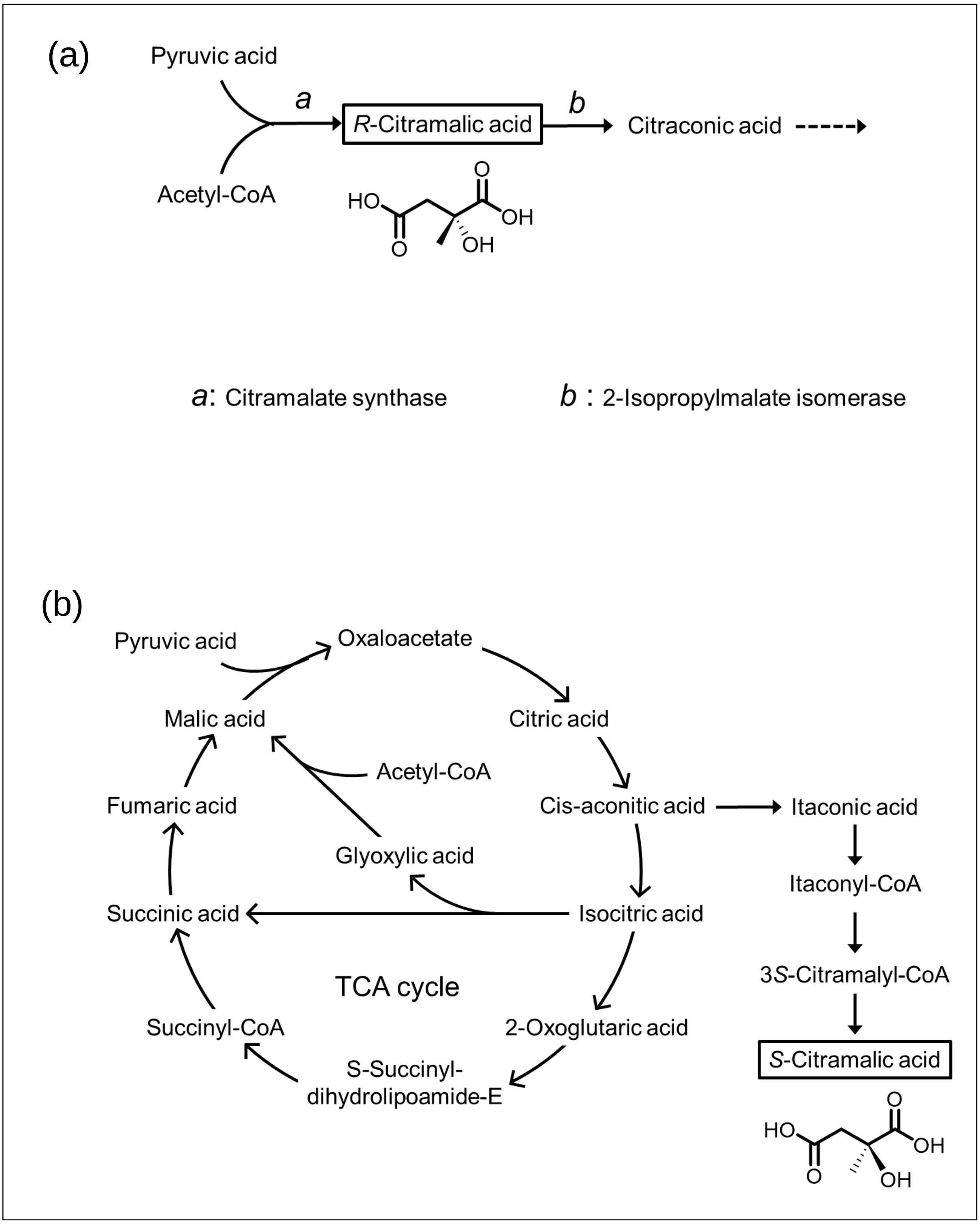

Scheme S1. Biosynthetic pathways for (a) *R*- and (b) *S*-CMA.

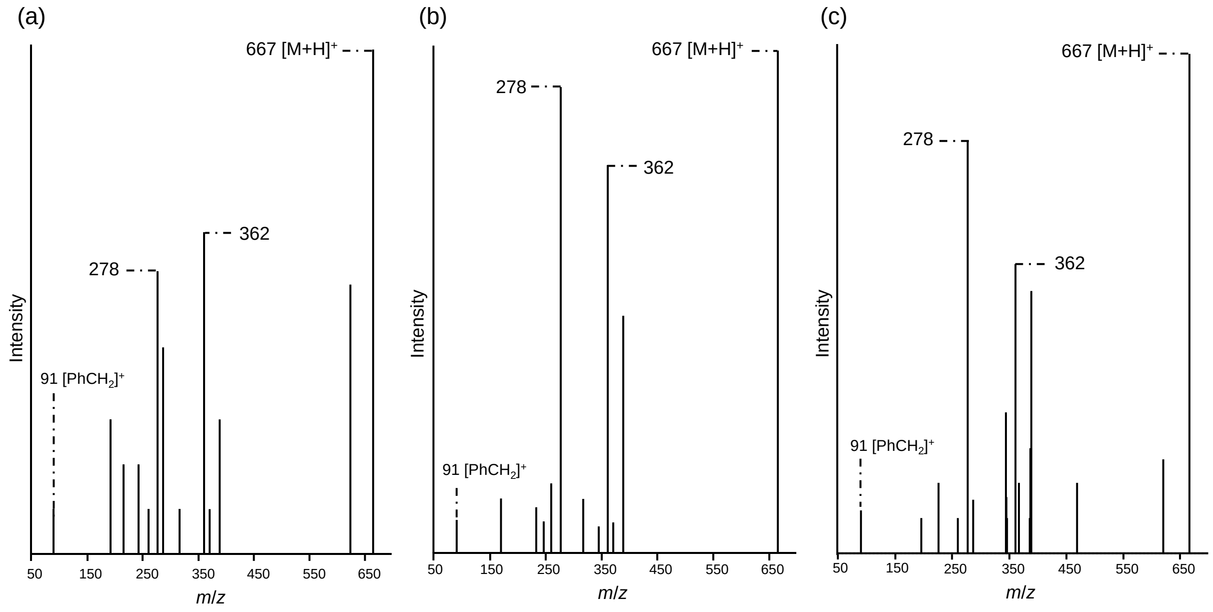

Figure S1. MS/MS spectra of *R*-CMA detected in the samples prepared from apple peel (a), fruit (b), and juice (c).

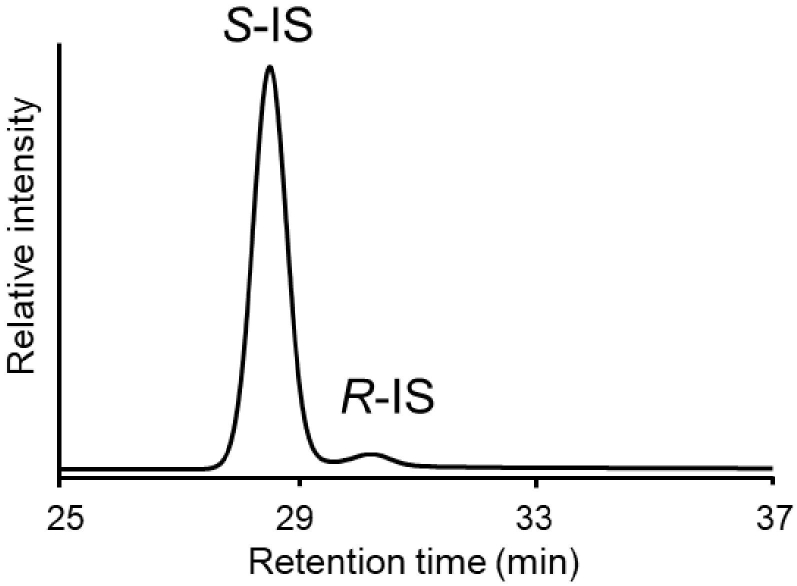

Figure S2. MRM chromatogram of the internal standard (10 µM sodium D-lactate ($^{13}C_3$, 98%) and 1.0 mM L-lactate -3,3,3-d_3) used in the present study.
